# Therapeutic potential of extracellular vesicles from diverse sources in cancer treatment

**DOI:** 10.1186/s40001-024-01937-x

**Published:** 2024-06-28

**Authors:** Haihong Lin, Jun Zhou, Tao Ding, Yifan Zhu, Lijuan Wang, Tianyu Zhong, Xiaoling Wang

**Affiliations:** 1 The First School of Clinical Medicine, Gannan Medical University, Ganzhou, 341000 China; 2 Laboratory Medicine, First Affiliated Hospital of Gannan Medical University, Ganzhou, 341000 China; 3 Department of Laboratory Medicine, Beijing Jishuitan Hospital Guizhou Hospital, Guiyang, 550000 China

**Keywords:** Cancer, Extracellular vesicles, Cancer therapy, Therapeutic vehicles, Engineered extracellular vesicles

## Abstract

Cancer, a prevalent and complex disease, presents a significant challenge to the medical community. It is characterized by irregular cell differentiation, excessive proliferation, uncontrolled growth, invasion of nearby tissues, and spread to distant organs. Its progression involves a complex interplay of several elements and processes. Extracellular vesicles (EVs) serve as critical intermediaries in intercellular communication, transporting critical molecules such as lipids, RNA, membrane, and cytoplasmic proteins between cells. They significantly contribute to the progression, development, and dissemination of primary tumors by facilitating the exchange of information and transmitting signals that regulate tumor growth and metastasis. However, EVs do not have a singular impact on cancer; instead, they play a multifaceted dual role. Under specific circumstances, they can impede tumor growth and influence cancer by delivering oncogenic factors or triggering an immune response. Furthermore, EVs from different sources demonstrate distinct advantages in inhibiting cancer. This research examines the biological characteristics of EVs and their involvement in cancer development to establish a theoretical foundation for better understanding the connection between EVs and cancer. Here, we discuss the potential of EVs from various sources in cancer therapy, as well as the current status and future prospects of engineered EVs in developing more effective cancer treatments.

## Introduction

Over the past few decades, scientific inquiry has increasingly revealed the vital role of EVs as conduits for communication between cells and their microenvironment. In the context of tumors, the function of EVs has garnered broad interest. They participate in biological processes such as angiogenesis and epithelial–mesenchymal transition, promoting tumor invasion and metastasis, and facilitate immune escape mechanisms, enabling tumor cells to evade immune surveillance. Additionally EVs significantly contribute to anticancer drug resistance, complicating treatment responses. However, it is encouraging to note that EVs from different sources have demonstrated diverse effects on tumor, potentially inhibiting tumor development. For instance, mesenchymal stem cell-derived extracellular vesicles (MSC-EVs) display considerable anti-tumor capabilities by inhibiting tumor angiogenesis, modulating cancer cell resistance to treatment, and regulating the immune response to suppress tumor growth. NK cell-derived EVs (NK-EVs) utilize multiple mechanisms to combat cancer cells effectively and are closely associated with immune surveillance and host defense. Tumor-derived EVs (Tumor-EVs) mediate anti-tumor activity by modulating macrophage phenotype and displaying high targeting specificity. Natural nanoscale particles, known as plant-derived EVs (Plant-EVs), facilitate precise intercellular material transfer and information exchange. EVs derived from gut bacteria (GB-EVs) significantly influence the intestinal barrier, immune response, and gastrointestinal health. In contrast, adipose tissue-derived EVs (Adipose tissue-EVs) are rich in anticancer miRNAs, inhibiting cancer cell growth and modulating cancer-related immune responses. In summary, EVs offer distinct advantages for cancer inhibition under specific conditions and show promise in therapy. Natural EVs effectively transport therapeutic molecules and chemotherapeutic agents. However, bioengineered EVs address limitations such as ineffectiveness and lack of targeting capability, enhancing therapeutic efficacy and minimizing adverse effects. This review aims to explore EVs’ role in tumor progression and therapy, offering insights for future cancer treatment strategies.

## Introduction to extracellular vesicles

Secretion of EVs is a fundamental and biological process, occurring across all cell types from bacteria to humans and in higher organisms [1, 2]. Encased in lipid bilayers, EVs lack a functioning nucleus and cannot replicate independently [3]. The “Minimum Information for Extracellular Vesicle Research 2023” document by the International Society for Extracellular Vesicles (ISEV) recommends using the generic term “EVs” and its extensions instead of ambiguous terms like “exosome”, which are linked to elusive biogenesis pathways [3]. Although EVs are commonly classified into two groups—large EVs (lEV, > 200 nm in diameter) and small EVs (sEV, < 200 nm in diameter)—it is important to distinguish between the terms “sEV” and “exosome”; the small EV population comprises both small exosomes and exosomes. Among the extensively studied and commonly used in tumor therapy are sEV [4]. sEV, also known as intraluminal vesicles, form through inward outgrowth of early endosomal limiting membranes, gradually maturing into multivesicular bodies [5–7]. Early endosomes (originating from the inward outgrowth of cytoplasmic membranes) and MVBs are involved in cellular material endocytosis and transport, particularly in sorting proteins for release [8].

EVs play essential roles in biological processes [9], including maturation of reticulocytes, cellular information transfer, immunomodulation, synaptic activity by regulating neurotransmitter release [10, 11]. They also influence tumor progression, either promoting or suppressing [12, 13]. Therefore, EVs have been investigated for applications in cancer therapy. For example, EVs have garnered attention for their potential applications in cancer therapy. They have been utilized as carriers for delivering drugs or therapeutic molecules to tumor cells [14], regulating sEV for cancer therapy [15], developing vaccines [16], and serving as diagnostic markers [17] (Table 1).

**Table 1:** Characterization of EVs subgroup

Features	sEV	lEV (formerly known as microvesicles)	References
Measurement	< 200 nm	> 200 nm	[3]
Biological features	Growth from early endosomes to late endosomes or multivesicular vesicles, released from the late endosomal compartment by fusion of multivesicular vesicles with the plasma membrane	Results of dynamic interactions between phospholipid redistribution and cytoskeletal protein contraction	[18–20]

## Involvement of EVs in cancer pathogenesis

**Fig. 1:**
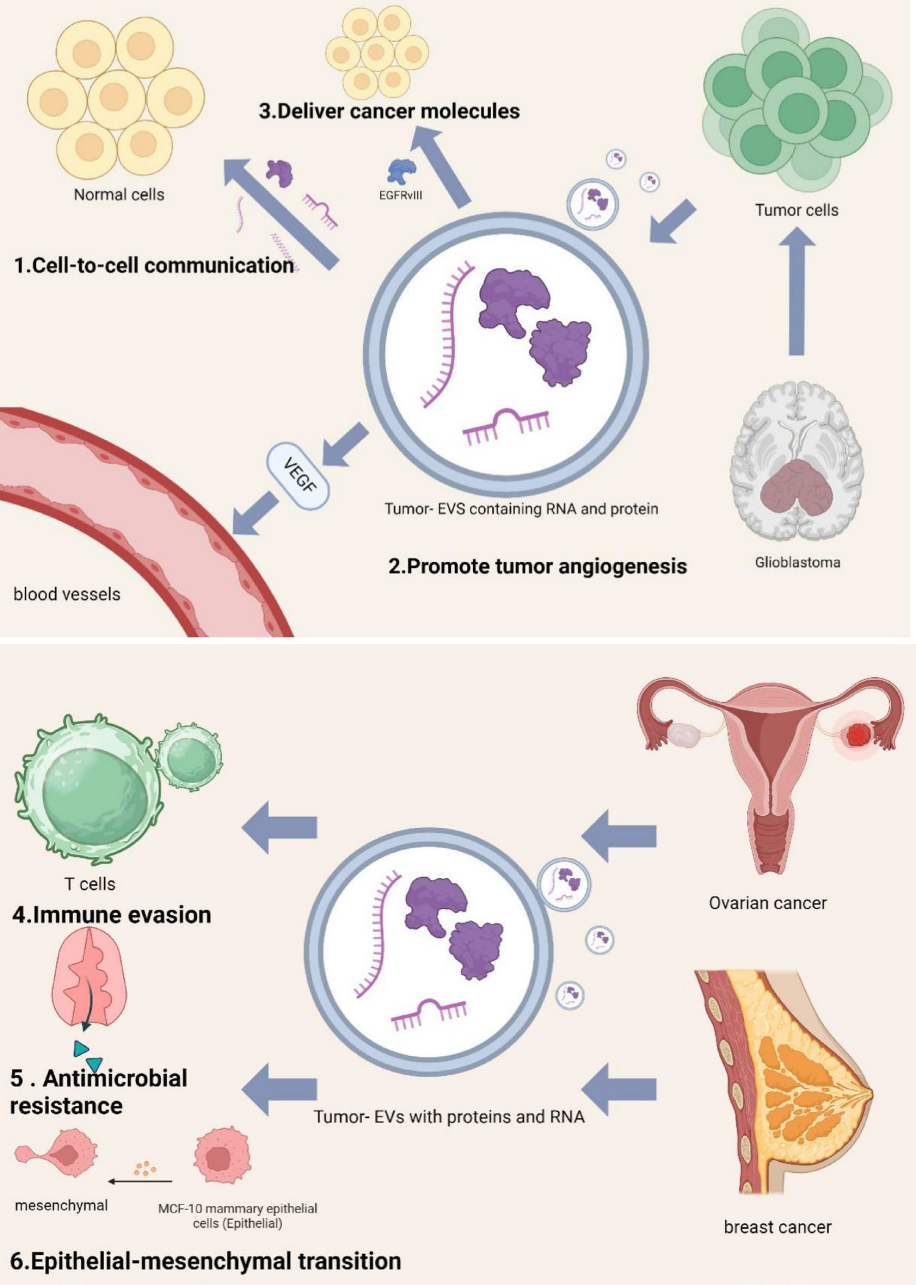
EVs are involved in cancer pathogenesis. (1) cell-to-cell communication: the contents of EVs can be transported from parental cells to neighboring cells. (2) Promotes tumor angiogenesis: EVs induce angiogenesis by stimulating endothelial cells to secrete cytokines and growth factors. (3) EGFRvIII can be transferred via EVs. (4) Immune evasion: enhances the proliferation and activity of regulatory T cells. (5) Antimicrobial resistance: EVs contain a variety of drug efflux pumps. (6) EMT: induces EMT in MCF-10 mammary epithelial cells

As crucial intermediaries of intercellular communication within the TME, EVs are endowed with copious bioinformatics that encompass proteins, small molecule metabolites, and lipids [21]. These chemicals have a multifaceted and crucial function in the tumor microenvironment, promoting cell maturation and significantly enhancing cell invasion and metastasis [21, 22]. EVs also promote various aspects of cancer progression, including angiogenesis [22] and anticancer resistance [23], and aiding tumor cells in evading detection by the immune system [24]. Thus, EVs play a vital role in both primary tumor progression and metastasis [25], underscoring their significance in cancer pathogenesis (Fig. 1).

### Transmission of cancer-causing molecules

Metastasis, an intricate biological phenomenon, encompasses a sequence of logical progressions facilitating the detachment of malignant cells from primary cell site and their dispersion to distant tissue. Upon their introduction into the novel microenvironment, these cells possess the ability to proliferate and initiate the development of ectopic lesions [26]. Electric vesicles are crucial in cancer metastasis by mediating changes in the physical and chemical properties of the TME and serving as important messengers in cell communication. EVs freely traverse the TME, significantly influencing tumor cell spread by creating a pre-metastatic niche (PMN), modulating specific signaling pathways, and promoting blood vessel formation [27, 28]. The PMN is a prearranged microenvironment created by cancer cells before the arrival of circulating tumor cells, facilitate tumor cell proliferation in other organs. Both tumor-secreted substances and EVs contribute to PMN formation [29, 30]. Studies have shown that EVs with miR-100-5p, miR-21-5p, and miR-139-5p from prostate cancer cells alter the PMN by enhancing metalloproteinase production and fibroblast migration [31]. Pei et al. showed in cellular tests that miR-203a-3p in extracellular vesicles is linked to macrophage polarization, promoting M2 polarization by reducing PTEN gene expression and stimulating the PI3K/AKT signaling pathway. Polarized M2 macrophages release CXCL12, which enhances tumor spread and facilitates the development of pmn in liver metastases of colorectal cancer through the CXCL12/CXCR4/NF-κB pathway [32].

EVs are capable of delivering oncogenic molecules between tumor cells. For example, Tumor-EVs containing EGFRvIII, produced by cells expressing epidermal growth factor receptor variant III (EGFRvIII), transmit these molecules to EGFRvIII-negative tumor cells within the same tumor [33]. Following receptor cell/tissue uptake mediated by Tumor-EVs, EGFRvIII triggers mitogen-activated protein kinase and protein kinase B signaling pathways, causing morphological alterations and promoting malignancy [33]. EGFRvIII can be transmitted by EVs from EGFRvIII-positive glioblastoma (GBM) cells to negative GBM cells, according to research by Al-Nedwi et al. [33]. EVs facilitate intracellular interactions between malignant and less invasive tumor cells across various cancer types. Highly metastatic breast cancer cells spread EVs containing the miR-200 family to non-metastatic breast cancer cells, thereby promoting lung metastasis, as demonstrated by Le et al. [34]. Qu et al. discovered that EVs also transport long-stranded noncoding RNAs, contributing to chemotherapy resistance in renal cell carcinoma cells [35]. Subpopulations of melanoma cells expressing high levels of the Met gene exhibit different phenotypes, increased resistance to BRAF inhibitors, and enhanced lung metastasis [36]. Met produced by Tumor-EVs originates from Met-high tumor cells, and enhanced Met expression in Met-low tumor cells maintains their metastatic potential in the lung [36].

### Extracellular matrix (ECM) remodeling

The extracellular matrix (ECM) is crucial for cell proliferation, motility, and differentiation. Comprised mainly of collagen, laminin, fibronectin, and proteoglycan, the ECM forms a robust physical network through non-covalent connections [37]. ECM remodeling is pivotal in cancer, creating pre-metastatic niches that impact tumor development, invasion, and dissemination. Abnormal expression of MMPs leads to ECM degradation, closely linked to cancer progression [38].

Tumor cells release EVs capable of modulating the structure and function of the ECM, ultimately promoting tumor spread. These EVs carry ECM-degrading enzymes like MMPS. Research has demonstrated that when highly metastatic MDA-MB-231 breast cancer cells transfer their EVs to non-tumorigenic MCF-10A epithelial breast cancer cells, MMP-2 and MMP-9 expression is upregulated, facilitating EMT development [39]. Additionally, sEV secreted by lung cancer cells contain lnc-MMP2-2, which enhances MMP2 production, thereby influencing cell migration and invasion mechanisms [40].

### Communication role

Cellular communication refers to the transmission of a message from one cell to another, eliciting corresponding responses. EVs are considered significant contributors to intercellular communication [41], carrying a diverse range of proteins, including cytokines, messenger RNAs, lipids, and noncoding RNAs like miRNAs and lncRNAs [42]. Under both normal and abnormal circumstances, EVs contents released can be transferred from the donor to recipient cells [43, 44]. For instance, EVs from pancreatic cancer cells expressing tetra-transmembrane protein 8 selectively attract proteins and mRNAs, activating angiogenesis-related genes in endothelial cells [45]. Glioblastoma-derived EVs, containing mRNA, miRNA, and angiogenic proteins, stimulate primary tumor growth and endothelial cells proliferation upon internalization by recipient cells [46]. Moreover, EVs contribute to tumor progression, as seen in breast cancer-associated fibroblast-secreted EVs, which promote breast cancer cell migration, infiltration, and dissemination by activating the Wnt-planar cell polarity signaling pathway [47]. As a result, EVs serve as the major effectors of the dynamic signaling circuit shaping the tumor microenvironment, facilitating bidirectional communication between tumor cells and their surroundings [25].

### Promote tumor angiogenesis

EVs play a role in cancer progression by promoting tumor angiogenesis, the formation of new blood vessels from existing ones to nourish tumor cells, aiding in their survival, growth, and dissemination [48]. This complex process includes multiple cell types and intricate signaling pathways [49], encompassing two primary mechanisms: sprouting angiogenesis, where endothelial cells create buds in response to angiogenic signals, and intussusceptive angiogenesis, involving the penetration of existing vessels by mesenchymal tissues and the creation of transmural vascular columns [50].

To initiate angiogenesis and advance cancer, tumor cells secrete EVs containing particular molecules such as miRNAs and lncRNAs [48]. For instance, EVs that generate nasopharyngeal carcinoma-producing microRNA-23a inhibit the anti-angiogenic factor TSGA10, promoting angiogenesis [51]. Elevated miRNA-23a expression enhances the pro-angiogenic effects of EVs in vitro and in vivo [52, 53]. Similarly, EVs from oxygen-deprived lung cancer cells induce the transformation of M2 macrophages through the miR-103a/PTEN pathway. M2-polarized macrophages release cytokines that promote cancer progression and angiogenesis [54]. Vascular endothelial growth factor (VEGF) is a group of soluble growth factors crucial in angiogenesis [55]. VEGF variants bind to endothelial cell receptors, inducing migration and proliferation, resulting in the development of new blood vessels. There is a speculation that cancer cell-derived EVs may facilitate this mechanism [56, 57]; for example, Al-Nedawi et al. demonstrated that EVs from A431 epidermoid carcinoma cells possess epidermal growth factor (EGF) receptors that [56], upon transfer to recipient cells, trigger signaling cascades promoting VEGF synthesis and angiogenesis [56]. Similarly, EVs from extremely aggressive glioblastoma multiforme cells trigger angiogenesis by stimulating endothelial cells to secrete cytokines and growth factors, facilitating the migration of pericytes [58].

### Immune escape

The TME harbors various immune cell types, such as lymphocytes, dendritic cells, monocytes, myeloid-derived suppressor cells, and granulocytes. These cells play a critical role in maintaining immunological surveillance [59]. However, tumor cells have the ability to efficiently alter signaling pathways within immune cells, converting them into immunosuppressive entities, thus promoting the survival and spread of malignant cells [60]. Studies have shown that Tumor-EVs trigger immunosuppression by facilitating the programmed cell death in hematopoietic stem cells (HSC), dendritic cells (DC), and peripheral blood lymphocytes (PBLC) [61]. Several Tumor-EVs are rich in Fas ligands, which induce apoptosis upon binding to their receptors. Fas-Ligands(+) Tumor-EVs promote regulatory T cell proliferation and anti-tumor T cell apoptosis, enabling evasion of the immune response [62–66]. Additionally, ovarian cancer cells produce EVs capable of stimulating the growth and activation of regulatory T lymphocytes [67]. Conversely, EVs from tumor cell lines can induce Fas-Ligands or tumor necrosis factor-associated apoptosis-induced ligand-dependent cell death in CD8 T cells [64, 65]. Tumor-derived miRNAs are also contribute to immunosuppression. For example, miR-214, transported by Tumor-EVs is transferred into recipient T cells. In vivo studies demonstrated that miR-214 promotes regulatory T cell expansion, leading to immunosuppression and increased tumor formation [68].

### Participation in epithelial-to-mesenchymal transition

EMT, a physiological process, occurs when epithelial cells transition into mesenchymal cells. This transformation commonly takes place during tissue healing, embryonic development, and tumor invasion and metastasis. The role of the EMT program in cancer has been extensively investigated, particularly in relation to its impact on the ability of cancer cells to undergo various stages of the invasive-metastatic process. This process involves the tumor cells invasion into nearby tissues, their uptake into the blood vessels, extravasation into distant tissues, and survival as small cancer cell deposits [69, 70]. EVs may also participate in the process of EMT. Ratajczak et al. discovered that EVs formed from embryonic stem cells enhanced the differentiation of target cells by stimulating the production of certain proteins associated with early pluripotent stem cells (Oct-4, Nanog, and Rex-1) and early hematopoietic stem cells (Scl, HoxB4, and GATA 2) [71]. Mesenchymal stem cell-derived EVs transport RNA to damaged renal tubular cells, resulting in changes in genetic expression and triggering differentiation [72]. Emerging evidence indicates that EVs generated from stem cells include biologically active substances, such as transcription factors and other genetic material, capable of promoting target cell survival and inducing a stem cell-like state [73]. Glioblastomas are the most prevalent malignant brain tumors originating within the brain. They can be categorized into three distinct molecular subtypes, with some degree of flexibility between them. Since pre-glioblastoma stem cell-like cells have the ability to change into the more aggressive mesenchymal subtype. Studies have demonstrated that EVs from mesenchymal congener cells can trigger mesenchymal transformation by activating the NF-κB/STAT3 signaling pathway. This leads to the development of cultured preneuronal cells exhibiting enhanced stemness, cell proliferation, migratory capacity, invasiveness, aggressiveness, and resistance to therapeutic interventions [74]. EVs produced by MDA-MB-231 breast carcinoma cells induced epithelial–mesenchymal transition in MCF-10 breast epithelial cells [75].

### Anticancer resistance

Recent years have witnessed significant advancements in cancer treatment. However, drug resistance remains a formidable challenge, leading to treatment failure and contributing significantly to cancer-related mortality [76]. Drug resistance is influenced by various intrinsic and extrinsic variables, including genetic and phenotypic alterations in cancer cells or surrounding cells, which can hinder drug absorption and facilitate drug expulsion, thereby reducing therapy efficacy [77]. Resistance can arise through horizontal transfer, where resistance is transmitted from resistant cells to susceptible ones [78]. One of the main obstacles to treating cancer is multidrug resistance (MDR), primarily linked to elevated expression of drug efflux pumps [76]. Several studies have demonstrated that EVs possess several drug efflux pumps, namely the ATP-binding cassette superfamily of transporter proteins, including P-glycoprotein, ABC transporter protein G2, and multidrug-resistance protein 1, facilitating the active removal of drugs [79]. Additionally, EVs harbor functional proteins implicated in drug resistance development. For instance, Ma et al. provided evidence that breast cancer cells resistant to the drug DOX exhibit increased levels of transient receptor potential channel 5 (TrpC5), a protein involved in generating electrical signals and allowing calcium ion passage. Furthermore, TrpC5 accumulates in the EVs released by these cells. TrpC5 through EVs allows receptor cells to acquire Ca2+ permeable channels, activating P-glycoproteins production and conferring chemotherapy resistance to non-resistant cells [76]. Furthermore, EVs possess a diverse range of noncoding RNAs, such as miRNAs, long noncoding RNAs, and cyclic RNAs. Mounting data indicate that the transfer of noncoding RNAs through EVs significantly influences the development of treatment resistance in cancer [80].

It is imperative to underscore the intricate nature of tumorigenesis, which encompasses numerous factors, genes, and phases. While EVs significantly contribute to this process, it is imperative to thoroughly examine additional factors such as genetic, physical, chemical, and viral influences. These elements contribute to tumor formation and thus require comprehensive investigation and analysis (Table 2).

**Table 2:** Involvement of EVs in tumorigenesis mechanisms

Involvement of EVs in tumorigenesis	Illnesses	Main mechanisms	References
Transmission of cancer-causing molecules	Melanoma	Increased resistance to BRAF inhibitors and lung metastasis by high levels of the Met gene	[36, 81, 82]
	Glioblastoma	EGFRvIII can metastasize through EVs	[33]
	Lymphoma	EVs containing miR-200 family can be transferred to non-metastatic breast cancer cells	[34]
ECM	Lung cancer	Upregulation of MMP2 expression affects ECM remodeling and tumor progression	[40]
Communications role	Pancreatic	EVs recruit proteins and mRNAs to activate angiogenesis-related gene expression in endothelial cells	[41, 45]
	Glioblastoma	Derived EVs promote primary tumor growth	[46]
	Lymphoma	EVs promote cancer spread through the Wnt-planar cell polarity signaling pathway	[47]
Promotion of tumor angiogenesis	Glioblastoma	EVs induce angiogenesis by stimulating endothelial cells to secrete cytokines and growth factors	[58, 83]
	A431 epidermoid carcinoma	Derived EVs contain EGF receptors that promote VEGF production and induce angiogenesis by stimulating signaling	[56]
Immune escape	Ovaries	Enhanced proliferation and activity of regulatory T cells	[59, 60, 67]
Promoting epithelial–mesenchymal transition	Glioblastoma	Induction of mesenchymal transition through NF-κB/STAT3 signaling	[58, 82]
	Lymphoma	Induction of EMT in MCF-10 mammary epithelial cells	[75]
Anticancer resistance	Lymphoma	EVs contain multiple drug efflux pumps	[76, 84]
	Nasopharyngeal carcinoma	Endoplasmic reticulum protein 44-containing EVs can translocate to neighboring cells to enhance chemoresistance	[85]

## Therapeutic potential of different sources of EVs in cancer

EVs, as key mediators of intercellular communication, play an important driving role in the process of tumorigenesis, progression, and metastasis by delivering oncogenic molecules, promoting tumor angiogenesis, inducing ECM remodeling, assisting immune escape, and participating in EMT. However, recent studies have gradually revealed that the effects of EVs on cancer are not solely promotional; they exhibit a complex and multifaceted dual role.

In addition to their detrimental carcinogenic capabilities, EVs demonstrate favorable anticancer properties. They are transporters of genetic material, such as microRNAs, pro-apoptotic factors, and anti-inflammatory cytokines, which inhibit the growth, progression, migration, and invasion of tumor cells, displaying tumor-suppressant properties [86]. Moreover, EVs are critical participants in intercellular immune system communication, conducting antigen presentation, immunosuppression, and polarization or activation of T cells and NK cells [87]. For example, sEV derived from dendritic cells stimulate the proliferation of NK cells dependent on IL-15Ralpha and NKG2D ligands, enhancing their tumor-killing capability [88]. EVs laden with a wide variety of substances not only function as biomarkers for cancer detection, but also hold promise as nanoscale drug delivery vehicles. Their remarkable drug delivery capabilities position them as vital components of drug delivery systems [89]. Even more encouragingly, EVs can be engineered as a potent synergistic agent in conjunction with anticancer medications, vaccines, or formulations. Through combined application, EVs can generate formidable synergistic therapeutic effects, opening avenues for novel disease treatment approaches. This article explores the therapeutic potential of various EV sources against cancer.

### MSC-EVs

MSC have been identified to perform various functions including angiogenesis, anti-apoptosis, anti-inflammatory responses, and immunomodulation [90]. EVs derived from MSCs have garnered growing interest in recent years as non-cellular alternatives to MSC-based therapies, showcasing therapeutic and tumor suppressive properties.

MSC-EVs have inhibitory effects on blood vessel formation. Zhu et al. were the first to document that EVs released by MSCs can actually enhance tumor development in live organisms [91]. Conversely, breast cancer cells present a different scenario. According to Lee et al., small sEV produced from human bone marrow stromal stem cells (hBMSC) inhibit the formation of new blood vessels and tumor progression both in laboratory settings and in living organisms. This is achieved by delivering a specific molecule called miR-16 to the tumor cells, which specifically targets VEGF and reduces its levels in breast cancer cells. Pakravan et al. observed that miR-100 is abundant in sEV produced from hBMSCs and inhibits vascularization in breast cancer cells by reducing the expression of VEGF in vitro [92].

Furthermore, miRNAs in MSC-EVs have tumor suppressor effects. As an illustration, the increase in miRNA-145 levels in sEV produced from human prenatal mesenchymal stem cells suppresses the progression of prostate cancer and triggers programmed cell death through the Caspase-3/7 pathway [93]. hBMSC-derived EVs can deliver miRNA-LET-7i to lung cancer cells, eliminating tumor cell proliferation through the lysine demethylase 3A/bicortin-like kinase 1/FXYD structural domain-containing ion transport regulator (KDM3A/DCLK1/FXYD3) axis [94]. Nevertheless, more comprehensive investigations are required to ascertain the probable pathways of sEV miRNA or other unidentified payloads in cancer advancement [95].

Additionally, MSC-EVs can modulate the cancer cell resistance to therapy, thus enhancing the therapeutic outcome. Application of MSC-EVs in a murine melanoma model demonstrated a significant enhancement in the effectiveness of radiotherapy by inhibiting tumor proliferation and metastasis [96]. In addition, the overexpression of miR-34c in MSC-EVs substantially increased radiation-induced apoptosis in nasopharyngeal carcinoma by inhibiting the β-linker protein, promoting apoptosis induced by radiation both in vitro and in vivo [97]. Furthermore, MSC-EVs can inhibit tumors by influencing the immune response. In breast cancer, MSC-EVs increase the expression of Programmed Death-1 (PD-L1) in myeloid cells and decrease PD-1 in T cells, suppressing the body's natural defense against tumors [98].

MSC-EVs exhibit superior safety and reduced immunogenicity compared to conventional cell-based therapies. The absence of cellular structure and function in these vesicles helps mitigate potential hazards such as uncontrolled cell proliferation and immune rejection. In contrast, the smaller size of MSC-EVs facilitates them to more efficiently traverse vessel walls and tissue interstitial space, thereby enhancing their ability to target and distribute substances. With ongoing advancements in preparation and purification technologies, comprehensive investigations into their biological properties and mechanisms of action, and continued progress in this field, MSC-EVs are expected to play a more significant role in future cancer therapies.

### NK-EVs

NK cells are essential immune cells that perform vital functions in immune surveillance and host defense against pathogens and tumors. They release biologically active molecules with cytotoxic properties, efficiently eradicating target cells [99]. NK-EVs, also known as sEV or MVs, are EVs generated by NK cells, primarily from peripheral blood monocytes and NK92 cells [100].

NK-EVs have a comparable behavior to memory, acquiring enduring characteristics upon exposure to cytokines or infections, known as immunological memory. These characteristics, present as cargoes of sEV or surface molecules, have the potential to enhance the cytotoxic activity of naïve NK cells, thereby improving defense function [100]. Multiple studies have proven the capacity of NK-EVs to target tumors specifically. Fluorescent signals were detected in tumors of a mouse glioblastoma xenograft model 92 h after administering sEV produced from NK-12 cells intravenously, with highest intensity seen at 48 h [101]. In an alternative mouse model of neuroblastoma, the utilization of sEV produced from NK-92 cells as a drug delivery system demonstrated effective targeting capabilities, evident from intense fluorescence noticed just 6 h post-injection. Furthermore, in mice with subcutaneous tumors, sEV were found in the tumor tissue within 20 min after treatment [102]. Nevertheless, the precise targeting mechanism of NK-EVs remains ambiguous. NK-EVs have the ability to transport cytotoxic proteins that specifically target and harm tumor cells. Both NK-92 cells and NK cells from human PBMC or mouse spleen can release sEV or MVs containing cytotoxic proteins. The type, quantity, and function of these proteins might differ based on the cellular source, physiological condition, or pretreatment. The cytotoxic effects of NK-EVs are not attributed to a single protein; instead, perforin, granzyme A and B, and granzyme hemolysin may all contribute to these effects [100]. NK-EVs carrying miRNA can suppress tumor cells, by transferring miR-155 and miR-21, which are produced by immune cells, to the tumor microenvironment. This delivery of miRNAs can have various regulatory effects on the target cells [103, 104]. Moreover, NK-EVs can serve as vehicles for delivering anticancer medicines or therapeutic compounds. As an illustration, paclitaxel (paclitaxel, PTX), a medication employed in the management of many types of malignancies, may be encapsulated within NK-EVs using electroporation. PTX-NK-EVs exhibited potent inhibitory effects on human breast cancer cells compared to an equivalent dosage of free PTX [105].

With their potent anti-tumor effects and unique characteristics, NK-EVs hold promise for cancer immunotherapy. They offer distinctive benefits and have the potential to be valuable additions to cancer immunotherapy. They can target and eliminate cancer cells through various ways and are tightly associated with immune surveillance and host defense. Future research will investigate the use of NK-EVs in cancer treatment, offering valuable insights for creating innovative and effective immunotherapies.

### Tumor-EVs

Tumor-EVs play a crucial role in cell-t-cell signaling and have promising applications in cancer treatment. Their advantages are manifold: firstly, Tumor-EVs effectively maintain the three-dimensional structure and cellular features of tissues, providing faithful representation of the pathophysiological traits and behaviors within TME [106]. This fidelity makes them invaluable for studying cancer development and potential treatments. Secondly, the Tumor-EVs exceptionally pristine due to its isolation from a single tissue [107, 108]. This characteristic significantly enhances the precision and reliability of the analytical findings. Ultimately, Tumor-EVs have the potential to regulate the TME, influencing tumor cell behavior and proliferation [109, 110], thereby presenting novel avenues for cancer therapy that hold promise for revolutionary advancements.

A multitude of studies have demonstrated that Tumor-EVs function via a diverse array of vectors and effectively regulate the phenotype of macrophages. While, M1 macrophages are linked to pro-tumor functions and M2 macrophages are implicated in anti-tumor activity [111]. However, Tumor-EVs possess a more potent targeting capability, which permits their modification to accommodate therapeutic agents. These agents can stimulate macrophages to impede cancer growth or eliminate tumor cells, thereby exerting anti-tumor and pro-inflammatory effects [112].

Considering their significant role in tumor development and metastasis, EVs represents valuable targets for new therapies. By blocking the formation, release, or absorption of EVs, it may be possible to disrupt the communication between CSCs and other cells within the tumor microenvironment, offering a new therapeutic approach [113]. For example, noncompetitive neutral sphingomyelinase inhibitors have been shown to reduce EVs secretion and reverse chemoresistance in colorectal, pancreatic and ovarian cancer cells [114–116]. Similarly, indomethacin is a potent anti-inflammatory drug, hindering lipid movement [117], has been found to sensitize lymphoma cells to chemotherapy [118]. In addition, hemofiltration has been suggested as a potential approach for eliminating tumor-derived extracellular vesicles from the bloodstream [119], while a novel approach utilizing aptamer-functionalized nanoparticles has been shown to prevent the infiltration of cancer-associated EVs into the small intestine and mitigate lung metastasis in a mouse model [120]. While these treatments have demonstrated efficacy in eliminating cancer-causing EVs from the bloodstream, more investigation is required to identify distinct oncogenic EV indicators for each kind of tumor [113].

### Plant-EVs

Comparable to the structural characteristics of animal EVs, plat-derived EVs share similar characteristics [121]. Plant-EVs are composed of lipid bilayers, surface membranes, and internal proteins, nucleic acids, and other things [122]. Furthermore, plant-EVs typically exhibit larger particle sizes compared to their animal counterparts [123]. These EVs originate from a diverse array of plants and can be extracted from many edible vegetables and fruits, playing crucial role in several physiological processes and facilitating intercellular communication [124–126].

Compared to the risk of cancer stimulation in animal EVs [127] and the harmfulness of artificial lipid carriers [128], Plant-EVs offer a high level of safety and cost-effectiveness. The lipid membranes of plant-EVs provide robust protection for bioactives against environmental elements, such as alterations in pH, heat, and light, while also demonstrating exceptional stability [129]. Plant-EVs possess intrinsic active substances from their source plants, endowing them with advantageous biological properties including antioxidant, anti-inflammatory, and anticancer effects [130]. With their potential to serve as efficient nanodelivery vehicles, plant EVs offer promising prospects for a wide range of cancer treatments. For example, a major challenge in the treatment of central nervous system disorders is the inability of drugs to penetrate the blood–brain barrier effectively. Many potential medications for brain tumors are underutilized due to their inability to cross this barrier at therapeutic levels [131]. To address this, Wang and his team effectively reassembled the lipids of grapefruit to form a nanocarrier derived from grapefruit. This approach facilitates the passage of therapeutic medications across the blood–brain barrier and, potentially enhancing the efficacy and stability of drug therapy for brain malignancies [130]. Plant-EVs, being naturally occurring nanoscale particles, possess the capability to transport molecules including lipids, nucleic acids, and proteins with precision and efficiency in intercellular communication and substance transfer. Thus, plant-EVs have the potential to be utilized as a drug delivery system for directly targeting tumor cells, thus enhancing therapeutic efficacy and reducing adverse effects [132].

### GB-EVs

There are hundreds of millions of microbiota residing in the intestine, which interact with the host and actively participating in metabolic processes. These microbiota play crucial roles in maintaining intestinal homeostasis, immune balance, and metabolism [133]. It has been demonstrated that GB-EVs significantly impact the intestinal barrier, immune response, and gastrointestinal health as a whole. By incorporating probiotics into one's diet or adopting a healthy dietary pattern, it is possible to effectively sustain the beneficial effects generated by the intestinal microbiota [134].

The equilibrium between host and commensal bacteria in the gut is crucial for maintaining a healthy human body. They work together to control the development and activity of intestinal epithelial cells and other immune cells. GB-EVs facilitate communication between hosts and pathogens, efficiently controlling the immune response and impacting the transmission of critical signaling pathways [135]. Furthermore, when small intestinal epithelial cells actively secrete EVs into the lamina propria, they relay information regarding orally administered antigens to immune cells, facilitating the modulation of the immune response via intercellular communication [136].

Additionally, GB-EVs support intestinal homeostasis, acknowledging the critical role of the epithelial layer in safeguarding the gastrointestinal tract against a wide range of toxins and pathogens is widely acknowledged. Studies have shown that EVs generated by both EcN and ECOR63 exhibit TcpC-mediated strengthening effects. These probiotics-derived extracellular vesicles were successful in upregulating the expression of ZO-1 and claudin-14 while decreasing the expression of claudin-2, leading to enhanced epithelial barrier function [137].

Bacterial extracellular vesicles contain a diverse range of RNAs that possess regulatory roles akin to miRNAs found in eukaryotic cells [138]. Hence, it is feasible that control of particular genes in recipient human cells can occur by the transfer of RNA molecules via extracellular vesicles. Speculation suggests that bacterial EVs might influence how human cells in the tumor microenvironment respond to cancer therapy [139].

GB-EVs show promise in cancer treatment, but additional investigation is required to understand their precise mechanisms and therapeutic benefits. Advancements in science and technology, along with extensive study, are expected to lead to more breakthroughs and discoveries in cancer therapy, offering new strategies and ways.

### Adipose tissue-EVs

Tumor tissue and adipose tissue are the two primary sources of Tissue-EVs in tumor diagnostic and treatment investigations, respectively. Adipokines, including pro- and anti-inflammatory and metabolic regulating cytokines, are secreted by adipose tissue, functioning as an endocrine organ [140, 141]. Since Tumor-EVs have already been thoroughly discussed, here we concentrate on adipose tissue-EVs.

Plentiful miRNAs have been found in Adipose tissue-EVs and have demonstrated anti-tumor properties [142–144]. For instance, Takahara showed that miR-134, previously known as a tumor suppressor, is found in EVs derived from human adipose tissue mesenchymal stem cells (ATMSC-EVs). This miRNA triggers apoptosis by enhancing the activity of B-cell lymphomas, thus suppressing the growth of prostate cancer cells [145]. EVs from healthy adipose tissue may create an environment that inhibits cancer cell proliferation, potentially enhancing cancer treatment and prognosis.

Adipose tissue-EVs can alter immunological responses related to cancer, potentially impacting the immune system and exerting an immunomodulatory influence on cancer. Human neutrophils internalize adipose tissue extracellular vesicles, reducing neutrophil cell death and enhancing their phagocytic ability. This process prompts neutrophils to shift towards the N1 type with increased phagocytic capacity, leading to decreased tumor growth [146]. This represents a promising approach for cancer treatment [144].

Studying extracellular vesicles released by adipose tissue in the process of tumor development can assist uncover novel targets to hinder tumor growth and offer creative approaches for tumor treatment [147]. Pharmacologically inhibiting fatty acid oxidation totally reversed the impact of adipose Tissue-EVs on the migration of obese tumor cells. Thus, FAO inhibitors have promise for use in anti-tumor therapy, particularly in obese individuals [148].

Adipose tissue-derived EVs have the capacity to convey more novel information and more accurately represent intercellular communication and metastasis than EVs derived from bodily fluids [149]. This makes adipose tissue-derived EVs a promising target for the advancement of novel therapeutic and diagnostic approaches towards tumors. The utilization of adipose tissue-EVs in the treatment of cancer is currently in the exploratory phase, requiring additional comprehensive investigation and refinement (Table 3).

**Table 3:** Advantages and disadvantages of different sources of EVs in cancer therapy

Sources of EVs	Advantages	Disadvantages	References
MSC-EVs	Mimicking parental cells with pro- and anti-tumorigenic effects and inherent tumorophilia	Controversial safety, high workload but low throughput in the production process	[95, 150, 151]
NK-EVs	Reversing immunosuppression under tolerogenic conditions and contributing to NK-mediated immune surveillance against tumors	Heterogeneity creates difficulties for dose determination and efficacy assessment	[100, 152]
Tumor-EVs	Involved in EMT and extracellular matrix remodeling to promote invasion and metastasis, contributes to the suppression of anti-tumor immune response by carrying different inhibitory molecules	Lack of insight into the content, spatial organization and heterogeneity of EVs	[153–156]
Plant-EVs	Low cost, more stable and can be used as natural nanocarriers in drug transportation systems	Challenges remain in the standardization of separation technologies for large-scale production and their efficient purification	[89, 130]
GB-EVs	Can effectively induce anti-tumor immune response in vivo, developed as a vaccine and used in combination with anticancer drugs	Uncertainty in targeting behavior after entry into host cells	[157, 158]
Adipose tissue-EVs	Carrying more raw information and a truer picture of intercellular metastasis and communication, a potential target for the development of new diagnostic and therapeutic approaches for tumors	Problems with preparation, dissemination, targeting and storage	[144, 149, 159]

## Overview of engineered extracellular vesicles

Cancer is a significant worldwide health issue, and it is expected that the global burden of cancer will keep rising over the next two decades. Its resistance to drugs, capacity for metastasis, and poor prognosis make it a formidable threat to human health, leading to high rates of morbidity and mortality. Despite significant advancements in healthcare technology, the clinical results of tumor therapy remain unsatisfactory due to the lack of precise medication targeting at the tumor sites, leading to suboptimal chemotherapy outcomes [160]. Multiple investigations and research have highlighted the potential of EVs as highly effective vehicles for drug delivery, offering opportunities to enhance the efficacy of cancer treatments [161]. Nevertheless, the utilization of natural EVs for tumor treatment is hindered by several constraints, including inadequate targeting capability and ineffective drug administration. Recently, efforts have been made to enhance the efficacy and targeting ability of EVs through engineering, unlocking their great potential in cancer therapy.

Engineering EVs involves using technology to modify and enhance their specific functions, such as improving targeting, regulating gene expression, serving as drug carriers, and modulating the tumor microenvironment, among others, to achieve specific application objectives [162]. Two essential stages comprise the preparation of engineered EVs: firstly, the extraction of EVs from cell culture supernatants; and secondly, the modification of EVs via genetic engineering and chemical modification. To endow extracellular vesicles with particular functions, genetic engineering combines the gene sequences of pertinent surface membrane proteins with ligands or homing peptides, while chemical modification improves the stability and targeting of EVs by introducing specific molecules onto their surface [163].

Engineered EVs have emerged as a highly innovative and effective modality in oncological therapeutics, providing a versatile platform for the delivery of anticancer drugs. These vesicles are proficient in encapsulating chemotherapeutic drugs and other bioactive molecules, facilitating targeted drug delivery and sustained release. This targeted approach enhances the therapeutic efficacy while minimizing systemic toxicity, thereby representing a significant advancement in cancer treatment paradigms [163]. Furthermore, engineered EVs can be synergistically combined with other treatment methods, such as photothermal therapy. This combination strategy aims to potentiate the antineoplastic effects across various malignancies by leveraging the thermal sensitivity of tumor cells. Such integrative therapeutic approaches not only enhance overall treatment efficacy, but also address the limitations inherent to monotherapies, providing a comprehensive and robust framework for cancer management [164].

### Limitations of engineered EVs

Engineered EVs offer precise targeting capabilities, enhancing the specificity of therapeutic interventions [165]. However several limitations persist in their technique. First, due to the varying biological properties of EVs secreted by different cell types, there is no standard protocol for their isolation. Consequently, combining multiple isolation techniques is necessary to improve efficiency and enable large-scale, standardized production of EVs [166]. Second, the diversity of EVs components contributes to their complex biological functions. It is important to elucidate the specific biological roles of different EV components and to further investigate the mechanisms of cargo loading and cellular targeting to enhance delivery efficiency and stability [113]. Although numerous animal experiments and clinical trials have shown that the injection of heterologous EVs does not induce significant toxicity to vital tissues, it is important to note that studies by Zhu et al. [167] and Mendt et al. [168] observed phenomena such as elevated neutrophil counts and tissue inflammation in mice injected with heterologous EVs. These findings suggest that heterologous EVs possess a certain degree of immunogenicity, necessitating a more rigorous safety evaluation. This evaluation should focus on assessing whether heterologous EVs might elicit immune and inflammatory responses in humans, thereby reducing the risk of unnecessary side effects. In addition, the regulatory framework and standards for engineered EVs are currently underdeveloped. This lack of robust regulations may lead to uncertainties during the research and development process, as well as pose significant regulatory issues in clinical applications. Establishing comprehensive guidelines and standards is essential to ensure the safe and effective use of engineered EVs in medical practice.

## Conclusion

In conclusion, EVs play a complex in cancer development. On one hand, they appear to establish a strong correlation with cancer, actively promoting cancer cell proliferation, aiding in immune evasion or elimination through pharmaceutical means, fostering a conducive environment for cancer metastasis, and significantly contributing to disease progression. However, on the other hand, EVs may inhibit tumor development by interfering with the normal functioning of cancer cells via specific mechanisms or by stimulating the immune system to attack the tumor. EVs derived from different sources have different advantages in inhibiting tumorigenesis, which in turn can be transformed into therapeutic targets. NK-EVs, for instance, have the capability of stimulating T-cells, macrophages, and other immune cells by secreting particular signaling molecules and antigens, orchestrating an attack against cancer cells. By employing this synergistic strategy, not only does it improve the body’s capacity to identify and eradicate cancer cells, but also enhances the treatment precision and efficacy. In cancer therapy, MSC-EVs serve as advantageous delivery vehicles due to their nanoparticle characteristics, potent tumor-targeting ability, minimal immunogenicity, and high tolerance.

Engineering modifications have substantially enhanced the targeting and drug delivery efficiencies of EVs, with their remarkable plasticity and robust loading capacity showing considerable promise in cancer therapy. Despite the promising potential of extracellular vesicle (EV)-based therapies, numerous challenges remain in both preclinical research and clinical application. Prior to the modification of EVs, a comprehensive understanding of the EVs membrane composition at the target site is crucial, along with the selection of appropriate targeting ligands. This approach is important to avoid changes in the EV membrane’s charge during the modification process and to maintain its physicochemical stability. Given that treated EVs may contain high level of impurities, stringent separation and purification protocols are imperative to ensure the safety of these therapies. Standardized guidelines for the isolation, purification, and toxicity testing of EVs is critical for their safe and effective therapeutic use. The most commonly employed methods for loading pharmaceuticals into EVs include incubation, electroporation, and sonication. Studies have demonstrated that sonication can achieve the loading efficiency of up to 29% with paclitaxel as a model drug, whereas incubation and electroporation achieve efficiencies of 1.5% and 5.3%, respectively. Enhancing the stability of delivery system and developing more efficient loading strategies thus represent significant challenges. Targeting EVs to specific cells, particularly within the complex and heterogeneous tumor microenvironment, remains a key issue. Engineering modifications to improve EV targeting while preserving membrane integrity is a critical area of focus. Most current research has been conducted in laboratory settings or using subcutaneous injections of cancer cells in immunocompromised mice. Notably, only a limited number of research have progressed to clinical trials to assess the effectiveness and safety EV-based therapies in cancer patients. This milestone is crucially necessary in the field, as it is through these studies that the scientific community can rigorously assess the therapeutic potential of EVs in cancer treatment. Conducting research in a clinical context will provide the necessary data to validate the efficacy and safety of EV-based therapies, ultimately paving the way for their potential integration into clinical practice. In-depth studies on the mechanism of action of EVs from different sources and precision engineering tailored to their specific properties will deepen our understanding of cancer occurrence and development. Moreover, this research may lead to the discovery of novel therapeutic avenues and methods in cancer treatment, injecting new vigor into the future development of medicine.

## Data Availability

Data availability is not applicable to this article as no new data were created or analyzed for this study.

## Abbreviations

EVs: Extracellular vesicle
MSC-EVs: Mesenchymal stem cells-EVs
NK-EVs: NK cells derived-EVs
Tumor-EVs: Tumor-derived EVs
Plant-EVs: Plant-derived EVs
GB-EVs: Gut bacteria derived-EVs
Adipose tissue-EVs: Adipose tissue derived-EVs
ISEV: International Society for Extracellular Vesicles
PMN: Pre-metastatic niche
EGFRvIII: Expression of epidermal growth factor receptor variant III
GBM: Glioblastoma
ECM: Extracellular matrix
EGF: Epidermal growth factor
VEGF: Vascular endothelial growth factor
HSC: Hematopoietic stem cells
DC: Dendritic cells
PBLC: Peripheral blood lymphocytes
MDR: Multidrug resistance
TrpC5: Transient receptor potential channel 5
PD-L1: Programmed death-1
hBMSC: Human bone marrow stromal stem cells
PTX: Paclitaxel
ATMSC-EVs: Adipose tissue mesenchymal stem cells
